# Collaborative discussions between GPs and pharmacists to optimise patient medication: a qualitative study within a UK primary care clinical trial

**DOI:** 10.3399/BJGP.2024.0190

**Published:** 2024-10-01

**Authors:** Roxanne M Parslow, Lorna J Duncan, Barbara Caddick, Carolyn A Chew-Graham, Katrina Turner, Rupert A Payne, Cindy Man, Bruce Guthrie, Peter S Blair, Deborah McCahon

**Affiliations:** Centre for Academic Primary Care (CAPC), Bristol Medical School, University of Bristol, Bristol.; Centre for Academic Primary Care (CAPC), Bristol Medical School, University of Bristol, Bristol.; Centre for Academic Primary Care (CAPC), Bristol Medical School, University of Bristol, Bristol.; School of Medicine, Keele University, Newcastle.; Centre for Academic Primary Care (CAPC), Bristol Medical School, University of Bristol, Bristol.; Department of Health and Community Science, University of Exeter, Exeter.; Centre for Academic Primary Care (CAPC), Bristol Medical School, University of Bristol, Bristol.; Old Medical School, University of Edinburgh, Edinburgh.; Bristol Medical School, University of Bristol, Bristol.; Centre for Academic Primary Care (CAPC), Bristol Medical School, University of Bristol, Bristol.

**Keywords:** general practice, general practitioners, medication reviews, medicines optimisation, pharmacists, polypharmacy, qualitative

## Abstract

**Background:**

There has been significant investment in pharmacists working in UK general practice to improve the effective and safe use of medicines. However, evidence of how to optimise collaboration between GPs and pharmacists in the context of polypharmacy (multiple medication) is lacking.

**Aim:**

To explore GP and pharmacist views and experiences of in-person, interprofessional collaborative discussions (IPCDs) as part of a complex intervention to optimise medication use for patients with polypharmacy in general practice.

**Design and setting:**

A mixed-method process evaluation embedded within the Improving Medicines use in People with Polypharmacy in Primary Care (IMPPP) trial conducted in Bristol and the West Midlands, between February 2021 and September 2023.

**Method:**

Audio-recordings of IPCDs between GPs and pharmacists, along with individual semi-structured interviews to explore their reflections on these discussions, were used. All recordings were transcribed verbatim and analysed thematically.

**Results:**

A total of 14 practices took part in the process evaluation from February 2022 to September 2023; 17 IPCD meetings were audio-recorded, discussing 30 patients (range 1–6 patients per meeting). In all, six GPs and 13 pharmacists were interviewed. The IPCD was highly valued by GPs and pharmacists who described benefits, including: strengthening their working relationship; gaining in confidence to manage more complex patients; and learning from each other. It was often challenging, however, to find time for the IPCDs.

**Conclusion:**

The model of IPCD used in this study provided protected time for GPs and pharmacists to work together to deliver whole-patient care, with both professions finding this beneficial. Protected time for interprofessional liaison and collaboration, and structured interventions may facilitate improved patient care.

## Introduction

Polypharmacy, the prescribing of multiple medicines to one individual, is increasingly common^1^ and can have a number of undesirable consequences, including increased risk of adverse drug effects, poor medication adherence, and a reduction in quality of life.^2^^–^^5^ There has been a shift from pharmaceutical care interventions, often led by pharmacists working in isolation, to more multidisciplinary interventions.^6^ Pharmacist-led interventions with better integration with the primary care team have been shown to be effective when focused on a small number of prescribing safety indicators in both trials^7^ and real-world roll out,^8^^,^^9^ but a key factor influencing implementation and mediating effectiveness is how pharmacists work with the wider primary care team.^10^^,^^11^

Medication review in primary care is key to addressing medicines optimisation and requires complex clinical decision making, balancing the risks from medicines with clinical benefit, while following evidence-based guidelines. There has been significant recent investment in pharmacists working in UK general practice to improve effective and safe use of medicines, in particular through the delivery of medication reviews.^12^ Better interprofessional working between GPs and pharmacists offers an opportunity during medication review to share clinical expertise and decision making in the context of therapeutic complexity and uncertainty, but evidence on how best to achieve this in the face of constrained resources is lacking.^13^

This study aims to report GP and pharmacist perspectives of undertaking interprofessional collaborative discussions (IPCDs) during a trial of a complex intervention to optimise medication use for patients with polypharmacy.^14^

**Table:** How this fits in

Pharmacists have a key role in addressing potentially problematic polypharmacy (multiple medications) in individual patients, but evidence of how this can effectively be done in collaboration with GPs is lacking. In this qualitative study, we found that pharmacists and GPs valued a scheduled, protected-time interprofessional collaborative discussion (IPCD). This strengthened their working relationship and provided a holistic approach to prescribing. Pharmacists felt the discussions gave them confidence to manage uncertainty and make independent deprescribing decisions in the future. General practices are becoming more multidisciplinary, and GPs and pharmacists could benefit from having IPCDs to support making joint, and potentially more informed, decisions about patient care and prescribing.

## Method

### Setting, design, and participants

The Improving Medicines use in People with Polypharmacy in Primary Care (IMPPP) trial was a large, cluster randomised trial that aimed to evaluate a complex intervention comprising a four-step structured process for medication review alongside performance feedback, financial incentives, and clinician training.^14^ The four-step review process comprised a case-note review carried out by the pharmacist, an IPCD between the pharmacist and GP, a review with the patient (pharmacist-or GP-delivered), and patient follow-up where clinically indicated. The IPCD was central to the medication review process and involved a 10-minute dedicated in-person meeting between the GP and pharmacist. The attendees discussed one or more patients to enable practitioners to share expertise and knowledge, and plan for the review with the patient. All participating GPs and pharmacists completed a comprehensive training programme to cover topics pertinent to the delivery of patient-centred polypharmacy medication review before commencing intervention delivery. The rationale for the IPCD and collaborative working was discussed during training alongside challenges to working together, solutions for overcoming barriers, and suggestions for how to undertake the IPCD in practice.^15^ Different models of pharmacist provision were used: pharmacists could already be working with a practice (practice or Primary Care Network [PCN]-employed) or be a ‘study pharmacist’ employed by the research team. Study pharmacists were not previously known to the practice they undertook reviews in. Each practice was supported by a unique pharmacist. Typically, one GP and pharmacist per practice were routinely involved in the intervention process.

Intervention practices were purposively sampled from all those taking part in the pilot/feasibility and full IMPPP trial to include different practice population sizes, levels of deprivation, models of pharmacist provision, and geographical areas. As part of a mixed-methods process evaluation, a detailed qualitative evaluation was undertaken in these practices, where participating GPs and pharmacists audio-recorded a subset of their IPCDs (ranging between 1 to 4 per practice) to understand their interaction and the utility of the discussion. Semi-structured interviews (see Supplementary Information for topic guide) were conducted by the first, second, and last authors with GPs and pharmacists separately, to explore their views and experiences of the trial and intervention. Interviews were held after a minimum of five trial medication reviews had been undertaken. Data collection stopped when practices had completed the intervention and when data saturation^16^ was achieved. In this article we report on practitioners’ use and views of the IPCD.

### Data analysis

Qualitative data analysis was ongoing and iterative, commencing soon after data collection.^17^ Verbatim transcripts from the audio-recorded IPCDs and interviews were uploaded into NVivo (version 11) and data analysed thematically, using a mixture of deductive and inductive coding, and adapted constant comparative techniques^18^ allowing the identification of both anticipated and emergent themes. Transcripts were read line by line for content and meaning, and a provisional coding framework outlined, with new codes added and existing codes merged to develop higher-level overarching themes. Analysis was led by the first author, who was the process evaluation researcher, with a subset independently double coded by the last author and compared to improve the reliability of the analysis.^19^ Themes identified were discussed within the qualitative research team (second, fourth to sixth and last authors) to agree the final coding framework. The interviews were analysed first, followed by the IPCDs to search for supporting evidence, adding to the coding framework.

## Results

Details of the 14 practices that took part in the process evaluation are shown in Table 1. A total of 17 IPCDs were audio-recorded across seven of these practices, discussing 30 patients. Multiple patients were usually reviewed and discussed as part of one IPCD; the length of IPCD audio-recordings ranged from 3 to 43 minutes depending on the number of patients recorded at a time (range 1–6 patients). In all, six GPs and 13 pharmacists (*n* = 5 practice, *n* = 5 PCN, and *n* = 3 study pharmacists) were interviewed across all 14 practices. Interviews took place in person (*n* = 1), by telephone (*n* = 5), or by video-call (*n* = 13), ranging in duration from 20 to 88 minutes.

**Table 1.** Intervention practices taking part in the qualitative evaluation

**Practice ID**	**Trial phase**	**List size, *n***	**Deprivation score^a^**	**Practice, PCN, or study pharmacist^b^**	**IPCD recordings, *n***	**Clinician interviews**	
						**GPs, *n***	**Pharmacists, *n***
A	Pilot	39 000	5	Practice	2	1	1
B	Pilot	14 000	9	Practice	2		1
C	Pilot	19 000	10	PCN	1		1
D	Full	14 000	10	PCN	4	1	1
E	Full	23 000	9	Practice	4	1	1
F	Full	16 000	9	PCN			1
G	Full	5000	6	PCN		1	
H	Full	9000	3	PCN			1
I	Full	13 000	5	Practice		1	1
J	Full	15 000	5	PCN	2	1	1
K	Full	5000	4	Practice	2		1
L	Full	6000	8	Study			1
M	Full	49 000	4	Study			1
N	Full	15 000	9	Study			1

a *Index of multiple deprivation decile for practice, 10 = least deprived.*

b *Practice pharmacists: already employed by practice; PCN: already employed by local primary care network; study pharmacist: external pharmacist provided by research programme. IPCD = interprofessional collaborative discussion. PCN = Primary Care Network.*

Emergent themes and subthemes are summarised in Box 1. Illustrative data are predominantly presented from the interviews, with data from the IPCDs (in Boxes 2–4) used to further illustrate themes identified.

**Box 1.** Themes and subthemes of healthcare professionals’ views of the benefits of interprofessional collaborative discussions

**Benefits of interprofessional collaborative discussions:** consolidates relationship between GP and pharmacist protected time allows discussion of the whole patient helps manage complex patients and uncertainty a different perspective enables joint learning improved knowledge and confidence of pharmacists — additional reassurance provided to patients
**Factors facilitating interprofessional collaborative discussions:** shared values around deprescribing
**Challenges of interprofessional collaborative discussions:** lack of time and wider practice engagement

**Box 2.** Joint decision making (IPCD audio transcript)

**Collaborative planning:**
GP20: *‘It’s highlighted that she is on an ACE* [angiotensin-converting enzyme] *inhibitor and an ARB* [angiotensin receptor blocker]*, which she is. So she’s on perindopril at quite a low dose of two milligrams. She’s also on candesartan and she’s on moxonidine.’*
PH21: *‘*[…] *she was discharged from that hospital with both candesartan and perindopril. So that’s what we need to review, I think.’*
GP20: *‘I agree. So, I would suggest that we discuss with her stopping her perindopril. Keeping a close eye on her blood pressure. She’ll obviously then just be on candesartan and moxonidine, but we have got scope to increase her dose of moxonidine …’*
PH21: *‘Moxonidine, yes.’*
GP20: *‘— further.’*
PH21: *‘Yes, but I would really suggest that we — because we’ve just increased, or the nurses have increased a* [over speaking 0:05:11.7]*. We need to give her about two weeks before we take her off the perindopril and then review again and then decide if the moxonidine can go a little bit higher and then take it from there really …’*
GP20: *‘Absolutely.’*
**GP suggesting lifestyle changes:**
GP18: *‘And then I suppose the other question is, what’s her diabetic control? Oh, it’s not terrible, is it, no. It’s been pretty steady really so she’s not on any medication, which I’m wondering why she’s not on metformin.’*
PH19: *‘She is, she is on metformin.’*
GP18: *‘And what’s her weight, she’s worried about her weight? I mean, she’s on metformin.’*
PH19: *‘BMI is thirty-seven.’*
GP18: *‘Would she consider the Lifestyle Club? If she lost a bit of weight that might help her blood pressure as well.’*

**Box 3.** Joint learning (IPCD audio transcript)

**Pharmacist sharing knowledge with GP:**
GP11: *‘What are they — edoxaban — is that the same for AF* [atrial fibrillation] *and DVT* [deep venous thrombosis] *and everything?’*
PH10: *‘Edoxaban for AF. You can use it to prevent DVTs, but you can’t use it to treat, like rivaroxaban.’*
GP11: *‘Okay.’*
PH10: *‘So if you’ve got someone new then you’re best sticking to rivaroxaban but if it’s AF, edoxaban should be first choice.’*
GP11: *‘But there’s so many things to know, and I know this is being recorded, that’s why I’m saying it. But it’s helpful revision just to go over guidelines and things, ’cause it’s quite complicated.’*
PH10: *‘Absolutely. Luckily, I do it all the time, so it’s fine. I don’t have to see loads of patients like you so it’s alright.’*
**GP sharing knowledge with pharmacist:**
GP18: *‘Did you say he’s on … because there seems to be aspirin still there.’*
PH19: *‘Well,* [name] *saw him after me, I mean he shouldn’t be on that should he?’*
GP18: *‘No, not if he’s starting on clopidogrel.’*

**Box 4.** GP knowledge of patients (from IPCD recordings)

PH19: *‘I’ll make a note for next time she comes in. She’ll probably come to see you. She was doing pretty well. She was taking the tablets; her blood pressure was a little bit high.’*
GP18: *‘It has been high for a long time, we’ve had lots of discussions about more medication, and does she want to take anything, and does she want to stop her HRT* [hormone replacement therapy]*, and she’s absolutely, “No, I know the risks, I’ll stick with it”, which is better than it used to be looking at that.’*

### Benefits of interprofessional collaborative discussions (IPCD)

GPs and pharmacists highly valued the IPCDs and described not only benefits but also some challenges.

#### Consolidates relationship between GP and pharmacist

GPs and pharmacists described the potential to feel isolated when working in primary care. When reflecting on usual care before the trial, GPs felt that pharmacists often worked separately, and pharmacists reported uncertainty about which GP to approach with medication queries in the busy primary care environment. Before the trial, communication was often *ad hoc* through electronic task messaging focused on single medication queries, often eliciting a ‘yes’ or ‘no’ response, and pharmacists described experiencing delays in receiving responses:

*‘I think the risk in general practice is sometimes a role can be quite separate and it’s* [IMPPP] *really helped us in bringing us in together.’*

(GP20)

Both pharmacists and GPs felt the IPCD brought them together and strengthened their relationship by getting to know each other and their roles, learning from each other, sharing values around deprescribing, and improved networking and communication. One pharmacist felt the trial enabled the GP to understand what they do. GPs described not necessarily working closely with pharmacy in usual care, whereas the trial enabled them to get to know their colleagues. The IPCD improved communication between practitioners and several GPs reflected that they now worked better as a team:

*‘It improved the communication, the camaraderie as well with the doctors.’*

(Pharmacist [PH] 16)

*‘It’s definitely helped me understand her* [pharmacist’s] *role but also, I think it’s been really nice working as a team.’*

(GP20)

*‘… It’s probably strengthened my working relationship with* [GP] *and I guess it’s good for her to see what we do as well.’*

(PH12)

#### Protected time allows discussion of the whole patient

GPs and pharmacists reported that the protected time afforded by the trial for the IPCD allowed two-way discussion as well as the opportunity to consider the whole patient, not just a simple medication query. Having a ‘dedicated’ GP was described as valuable because the pharmacist could check in with one person rather than having to identify someone to talk to within the practice. Protected time also allowed space for GPs and pharmacists to reflect on their own practice. One pharmacist described undertaking a more thorough review of patient notes and greater focus on deprescribing in preparation for the IPCD:

*‘… I think because when you do it in that electronic way, you tend to just deal with that issue and move on. It doesn’t open up necessarily a dialogue* […] *when you’re doing it in a more dedicated way with focusing on each individual patient and looking at their whole pharmacology, not just maybe that a single medication that’s been flagged up as an issue — you get a much better idea of what’s going on with the whole patient.’*

(GP5)

*‘… It made you look at it a bit more in terms of because you’re preparing for the interprofessional discussion you are looking for things probably more thoroughly to discuss …’*

(PH22)

Pharmacists reported coming to the IPCD meeting with notes or suggestions curated after assessment of the patient notes, and jointly discussing and reaching consensus with the GP on a plan to take to the subsequent review with the patient. Pharmacists and GPs described how GPs often had more in-depth knowledge of patients and the medication that might have been tried before. Some pharmacists took a leading role in the IPCD and felt GPs tended to have different insights into alternative strategies to try to reduce medication, including addressing lifestyle factors such as weight management (see IPCD audio transcript in Box 2):

*‘… generally we just come to a consensus view, so there’s never certainly been any disagreements.’*

(GP5)

*‘… she’s* [GP] *more experienced than I am, she’s been doing it for much longer, so some patients she knows, so if I’m saying you know, so I can make this change and she’ll say “Oh we’ve tried that already, this was the outcome so maybe let’s try a different way.”’*

(PH16)

*‘She* [GP] *tends to just go with what I’ve suggested because it’s things that she’s like, “I wouldn’t know they needed a DOAC* [direct oral anticoagulant] *dose review.”’*

(PH12)

The audio-recordings of the IPCDs (Box 2) supported the interview data showing how GPs and pharmacists had open discussions and reached consensus.

#### Helps manage complex patients and uncertainty

GPs and pharmacists described the IPCD as a highly valuable, helpful, and beneficial aspect of the IMPPP intervention. A more collaborative multidisciplinary team (MDT) approach was felt to be particularly important for patients with ongoing investigations or more problematic polypharmacy. This approach provided an opportunity to draw on knowledge, reflect and learn with colleagues, and to develop a plan before consultations with patients:

*‘For problematic patients try to keep the MDT approach to it. So, like there’s targeted ones that we have that are difficult, so problematic polypharmacy* […] *to try and have a proactive chat about that beforehand because they’re difficult conversations to have and it is genuinely quite helpful to have a pre-plan.’*

(PH14)

One GP felt that primary care is moving towards more multidisciplinary working and the IPCD was valuable for older patients with multimorbidity. Liaison with a colleague was particularly important when dealing with the clinical uncertainty that more complex patients bring. One pharmacist reported seeing benefits of the IPCD even after reviewing only a few patients. Some pharmacists wanted the IPCD to continue following the trial:

*‘So it’s that judgement and the grey bit between the black and white of medicine. There’s a lot of grey and I think it’s helpful to discuss the grey bits with other colleagues because it helps you navigate more …’*

(GP11)

*‘I definitely value the GP discussion. I guess that’s the main difference compared to what I’m doing in my day-to-day role and I have seen a benefit from it, even in the very small numbers that we’ve done.’*

(PH4)

#### A different perspective enables joint learning

GPs and pharmacists suggested that the IPCD enabled joint learning. GPs reflected that they respected pharmacists for their knowledge about drugs, including drug interactions, adherence, side effects, and current guidelines. Pharmacists felt GPs had a whole-person approach, and often raised things they had not considered. Some pharmacists felt they themselves had a more focused perspective compared with GPs, who looked at things more broadly and were more willing to suggest medication changes. The IPCD brought issues to the fore to ensure things were not missed:

*‘I think the most valuable thing is actually is that clinical discussion with someone who’s got a different perspective to me, who’s a pharmacist, who has a lot more time and knowledge about drugs …’*

(GP11)

*‘I get my tunnel vision on and* [GP] *is able to say step back a little bit.’*

(PH21)

*‘… that interprofessional conversation allows me to talk it through with someone else because otherwise you’re left to your own devices* […] *that’s where you make a mistake.’*

(PH16)

One pharmacist described the IPCD as a ‘very good learning event’ (PH4) and had altered decisions as a result of the discussions. One GP felt it allowed them to stay up to date with rapidly changing guidelines:

*‘I’ve found it* [IPCD] *really helpful* […] *having someone to discuss things with is actually* […] *‘cause guidelines and things are changing so rapidly it’s actually quite hard to* […] *say stay up to date.’*

(GP11)

*‘I was going to consider stopping amlodipine with the patient but actually when I spoke with GP, we decided to consider stopping his beta blocker* […] *I would say I changed my tack in terms of what I would’ve done had it just been me on my own without the GP.’*

(PH4)

Joint learning was evident in discussions between GPs and pharmacists in the audio-recordings of the IPCDs (Box 3). There was shared knowledge of medication risks, patients’ previously known preferences, and seeking each other’s opinions while discussing patients.

#### Improved knowledge and confidence of pharmacists

Pharmacists described how the IPCD gave them confidence in their decision making and interactions with patients. It provided an opportunity to check and discuss potential medication changes with the GP, giving pharmacists experience of different deprescribing scenarios, improving clinical knowledge and therefore confidence in dealing with the same issues with future patients. Pharmacists felt that GPs were often more brave or assertive in making changes. Pharmacists often brought suggestions to the meeting that were confirmed by the GP, giving them reassurance in their decision making:

*‘She* [GP] *was often a little bit more brave or assertive around changes, things that I wouldn’t probably have done which maybe in future will have given me a bit more confidence.’*

(PH19)

*‘… the conversation that* [GP name] *and I had was empowering for myself as well because he was happy with my comments and input. That brings a lot of confidence to me and what I’m doing.’*

(PH24)

One pharmacist described being more confident in making decisions without checking with a GP:

*‘… six months ago were impossible, changes that I wouldn’t have considered making without* […] *asking a doctor or asking someone to make a decision on, whereas I’m confident because I’ve seen it done multiple times and I can understand the reasoning behind it.’*

(PH16)

Another pharmacist (PH22) also described how the IPCD as part of the trial had made them more confident to approach a busy GP for a chat in the future.

#### Additional reassurance provided to patients

Pharmacists also described this confidence extending to their interaction with patients as they had already formed a ‘pre-plan’ with the GP. Pharmacists and GPs both reflected that GPs were more likely to know the patient’s previous care and preferences. Pharmacists therefore felt more certainty following the IPCD to recommend potential changes to the patient. Pharmacists often emphasised the multiprofessional element of the review to patients, because they felt this helped to increase patient confidence and trust in any suggested changes to medication. Study pharmacists, compared with practice/PCN pharmacists, were more likely to emphasise this in order to build rapport with patients. It was felt that, though patients were getting used to seeing allied health professionals, some patients still wanted to know that the GP had been involved. One pharmacist recounted a patient who felt strongly that the GP needed to be involved in any decision making. Others reported how patients appreciated the additional time pharmacists had to discuss their medicines. Some practices had pharmacists who had been undertaking medication reviews for many years and patients seemed used to talking to the pharmacist. Study pharmacists (as opposed to practice/PCN pharmacists) were more reliant on GPs’ knowledge of patients:

*‘You’re much more confident when you speak to the patients because I know what* [GP name] *thinks and then I can just follow that.’*

(PH14)

*‘I was just able to say “I’ve already spoken with your GP and we think it’s a good idea for you to start.”’*

(PH14)

*‘… particularly kind of elderly patients I think, they wanted to know that their own GP had been involved in this decision …’*

(GP20)

*‘… the patients have enjoyed the opportunities to really have a real sort of “deep dive” with me as a pharmacist with their medication.’*

(PH4)

There was evidence in the IPCD recordings (Box 4) that GPs knew several patients well from previous interactions, including the patients’ adherence to medication and previous efforts at exploring alternative medications and deprescribing, which helped inform decision making.

### Factors facilitating interprofessional collaborative discussions

#### Shared values around deprescribing

A supportive culture within the practice, and GPs with a positive opinion about pharmacists and their capabilities, seemed to facilitate more valuable collaborative discussions. Additionally, a lack of hierarchy, where pharmacists have equal standing to GPs and have shared values and ambitions around deprescribing, made working together and deprescribing decisions more cohesive:

*‘I think my practice, it’s the flattest sort of practice I’ve ever been in. And* [pharmacist] *is a partner like me* […] *I think the way to think about it is we’ve all got complementary skills and the world’s a complicated place and there’s not easy… not always an easy right or wrong answer. And often it’s a judgement and a discussion and I think he’s kind of leading the way and you know, making us think of the team and the wider MDT.’*

(GP11)

*‘The GP who was the lead on this project has shared some of the same ambitions as I do around medication reviews and deprescribing so I think we’ve got a common ground that meant that actually a lot of the conversations that I’d had were fitting in with her ideas as well.’*

(PH19)

### Challenges of interprofessional collaborative discussions

#### Lack of time and wider practice engagement

Pharmacists reported finding time to fit in the IPCD discussion to be challenging, particularly those working across multiple sites. Though the trial did not require the IPCD to be in person, most practices felt it was important to have an in-person meeting and this could be difficult to arrange. Some pharmacists and GPs felt that the IPCD was not as valuable for less complex patients and pharmacists felt confident that they could deal with these patients on their own in routine care (though this did not adversely impact trial fidelity):

*‘That’s the biggest restraint in general practice, isn’t it, is trying to fit it in and try and find time to do it* […] *coordinating across two sites was tricky because we decided to do it in person.’*

(GP15)

*‘Some of them* [patients] *were quite straightforward, which I’ll be able to manage myself.’*

(PH22)

One pharmacist felt the IPCDs were often rushed and the GP had not prepared for their discussion:

*‘… she’s* [GP] *good but it was often quite, I would say, a little bit rushed sometimes and there would be no preparation time on her behalf so it would be a bit like me presenting a case and study sort of thing and having feedback on it which was, you know, okay, but maybe it would have been different to see how she did it without my input.’*

(PH19)

In most cases, only one GP and pharmacist in each practice were routinely involved in intervention delivery. Several pharmacists reflected that it would have been useful to involve more colleagues and/or everyone in the practice. This may have helped wider team relationships and improved understanding and engagement with polypharmacy:

*‘… the main thing is having everyone engaged in the process, so sometimes I guess with the training it was two people from each practice so it’s probably really helpful like a prescribing masterclass session with everybody and, again, it helps just to engage people onto “this is what we’re trying to do” but it’s really hard with polypharmacy just because there’s like, I don’t know, 100 different indicators.’*

(PH14)

## Discussion

### Summary

This study identified that protected time with a named GP and pharmacist for an IPCD is valued and strengthened the working relationship. The IPCD helped to address uncertainty and to tackle the grey areas of medication management in more complex patients, with both professionals learning from each other and gaining confidence in decision making. GPs and pharmacists all reported that finding the time in a busy practice was challenging. Shared values around deprescribing within the practice facilitated collaborative working.

Primary care is becoming more multidisciplinary. The model of IPCD studied provided brief protected time for GPs and pharmacists to work together to deliver whole-patient care, with both professions finding this beneficial. Clearly defined roles for healthcare professionals, interprofessional collaboration, and structured interventions may support improved prescribing and thus patient care.

### Strengths and limitations

The comparison of both interviews and IPCD recordings allowed a more in-depth understanding of GP and pharmacist views of the discussions and what actually happened in practice. Involvement of more than one person in the analysis enhanced the trustworthiness and credibility of the interpretation and analysis.^20^^–^^22^

A number of limitations should be noted. Only six GPs were interviewed and recordings of IPCDs were only available from seven out of 14 practices taking part in the process evaluation. A wider availability of data from GPs and recordings of the discussions may have provided different findings. Additionally, data were gathered from practices located within just two regions of England where the IMPPP trial was being conducted and we were unable to recruit practices from the least deprived quintile to participate in the process evaluation, which may reduce the generalisability of the findings.

### Comparison with existing literature

A recent review revealed that pharmacist integration reduces potentially inappropriate prescribing in comparison with usual care.^6^ Most interviewees in this study were very positive about working collaboratively and described a number of benefits. This is similar to other studies that have found that GPs value pharmacist expertise.^22^^–^^24^ Like this study, a review of interprofessional collaboration between community pharmacists and GPs found that collaboration led to greater understanding of each other’s capabilities.^25^ A recent realist review suggests that clear roles for pharmacists facilitated closer communication and better relationships to improve multidisciplinary medication review for older people.^26^ Lack of time is a key barrier found in the literature,^13^ as was also found in this study. Even though we have described an activity undertaken in a trial environment, it is nevertheless reassuring that clinicians were able to accommodate the required 10-minute meeting around existing clinical workloads, suggesting the meeting should be feasible to deliver in real practice. The importance of increasing patient awareness of the role of practice pharmacists has also been previously identified.^27^ Our study highlights the potential benefits of informing patients of a multiprofessional approach to provision of care.

### Implications for research and practice

The NHS long-term workforce plan^28^ recommends the expansion of the roles and responsibilities of allied health professionals in primary care. This study has demonstrated that a dedicated IPCD can improve working relationships between GPs and pharmacists, and upskill pharmacists to be more confident in making future decisions. When the multiprofessional element of the review process was emphasised to patients, GPs and pharmacists reported that this seemed to increase patient trust in recommended medication changes.

The IMPPP trial allowed practices flexibility in terms of implementation of IPCDs, and we do not believe our data support one particular model. However, practices considering this approach may wish to consider the following points. To facilitate the delivery of improved collaboration between GPs and pharmacists, we recommend pairing pharmacists with a named GP and allow protected time for discussions. Regular scheduled meetings may help ensure delivery in practice. We recommend that IPCDs focus on complex patients to jointly explore areas of uncertainty and improve clinical decision-making skills of GPs and pharmacists. Focusing on complex patients, which pharmacists found particularly valuable, may also reduce workload and thus aid acceptability and adoption in routine practice. Multiple patients might be discussed in one IPCD meeting to ensure the meetings are time efficient and easier to organise. We also recommend communicating the multiprofessional approach to patients to increase their trust and engagement.

Because the IPCD forms only one part of the IMPPP complex intervention, future research might examine the independent impact of such interactions upon clinical outcomes, as well as quantifying patient and clinician experience. Additionally, further research might explore how the environment can be best optimised to support delivery of IPCDs, as well as how they should best be conducted in a clinical setting.

## Data

All data requests should be submitted to the corresponding author for consideration. Access to anonymised data may be granted following review via the University of Bristol Research Data Storage Facility.
